# A cleavage-based surrogate reporter for the evaluation of CRISPR–Cas9 cleavage efficiency

**DOI:** 10.1093/nar/gkab467

**Published:** 2021-06-04

**Authors:** Soo Bin Jung, Chae young Lee, Kwang-Ho Lee, Kyu Heo, Si Ho Choi

**Affiliations:** Research Center, Dongnam Institute of Radiological and Medical Sciences (DIRAMS), Busan, 46033, Republic of Korea; Research Center, Dongnam Institute of Radiological and Medical Sciences (DIRAMS), Busan, 46033, Republic of Korea; Center for Epigenetics, Van Andel Research Institute, Grand Rapids, MI, 49503, USA; Research Center, Dongnam Institute of Radiological and Medical Sciences (DIRAMS), Busan, 46033, Republic of Korea; Research Center, Dongnam Institute of Radiological and Medical Sciences (DIRAMS), Busan, 46033, Republic of Korea

## Abstract

CRISPR–Cas9 is a powerful tool for genome engineering, but its efficiency largely depends on guide RNA (gRNA). There are multiple methods available to evaluate the efficiency of gRNAs, including the T7E1 assay, surveyor nuclease assay, deep sequencing, and surrogate reporter systems. In the present study, we developed a cleavage-based surrogate that we have named the LacI-reporter to evaluate gRNA cleavage efficiency. The LacI repressor, under the control of the EF-1α promoter, represses luciferase or EGFP reporter expression by binding to the *lac* operator. Upon CRISPR–Cas9 cleavage at a target site located between the EF-1α promoter and the *lacI* gene, repressor expression is disrupted, thereby triggering luciferase or EGFP expression. Using this system, we can quantitate gRNA cleavage efficiency by assessing luciferase activity or EGFP expression. We found a strong positive correlation between the cleavage efficiency of gRNAs measured using this reporter and mutation frequency, measured using surveyor and deep sequencing. The genome-editing efficiency of gRNAs was validated in human liver organoids. Our LacI-reporter system provides a useful tool to select efficient gRNAs for genome editing.

## INTRODUCTION

CRISPR–Cas9 is an adaptive immune system that protects prokaryotes from invasion by foreign genetic elements (1–3). It consists of clustered regularly interspaced short palindromic repeats (CRISPR) and CRISPR-associated protein 9 (Cas9) (2–5). CRISPR RNA (crRNA), generally called guide RNA (gRNA), is responsible for target specificity by binding to a complementary target DNA sequence, and trans-activating crRNA (tracrRNA) acts as a scaffold that is recognized by a Cas9 enzyme. A single gRNA (sgRNA) containing both crRNA and tracrRNA directs Cas9 nuclease activity to the specific target of gRNA in the genome (1,6–8). The CRISPR–Cas9 system cleaves 3 bp upstream of the 5'-NGG-3' protospacer adjacent motif (PAM), which is required for Cas9 nuclease activity in the target sequence (7,9).

The CRISPR–Cas9 system is a powerful tool for genome editing. DNA double-strand breaks (DSB) caused by CRISPR–Cas9, together with mammalian repair systems, can produce gene deletions or substitutions of DNA sequences within desired genes. DNA DSBs produced by CRISPR–Cas9 can be repaired through two mechanisms: a non-homologous end-joining (NHEJ) or a homology-directed repair (HDR) process (10–16). NHEJ is a canonical homology-independent pathway for the religation of two ends, which results in random insertions or deletions of nucleotides. As a result, NHEJ repair can cause frameshifts, generating a nonsense mutation within the protein-coding region. In contrast to NHEJ, HDR is a more accurate repair mechanism and requires DNA templates to repair a damaged DNA strand. These templates can be provided in various forms, including sister chromatids, plasmids, or single-strand DNA oligonucleotides (ssODN) (10,17).

To improve gene editing efficiency with CRISPR–Cas9 systems, it is important to select an efficient 20 bp guide sequence that determines target specificity and cleavage efficiency. Many studies have reported the design of better gRNAs and the determination of the cleavage efficiency of these gRNAs, for example by *in silico* gRNA design (18–23), PCR (24), indel detection by amplicon analysis (IDAA) (25), surveyor assay (26,27), T7E1 (28–31), and deep sequencing. Although *in silico* gRNA design technology has greatly improved, experimental validation is still needed due to the complexity of the intracellular environment. Labuhn et al. showed that sgRNA-predictive algorithms and the measured activity of the generated sgRNAs are only slightly correlated, suggesting that a validation tool for sgRNA would be useful for selecting the most efficient sgRNAs (32). Multiple surrogate reporter systems have been developed to measure the efficiency of CRISPR–Cas9. One of these utilizes target sites for gRNA placed within the encoding region of a fluorescence reporter gene and evaluates gRNA efficiency by measuring the expression of fluorescence reporters (32,33). Another system, known as universal donor as reporter (UDAR), examines the efficiency of CRISPR–Cas9 in targeted mutagenesis based on the NHEJ-mediated knock-in strategy by measuring EGFP expression (34). CRISPR–Cas9-stimulated homology-directed repair has been evaluated using a transposable GFP reporter (35). Single-strand annealing (SSA)-based surrogate reporter expression and HDR reporters measure CRISPR editing efficiency and enrich genetically modified cells (36). Surrogate reporter systems are also used to enrich genetically modified cells by CRISPR–Cas9 mediated-NHEJ repair or CRISPR–Cas9-mediated HDR (37–40). Despite the recent improvements in these reporter systems, a simple and quantitative assay to measure the efficiencies of multiple gRNA candidates is still needed.

In the present study, we developed a novel cleavage-based surrogate reporter system, designated as LacI-reporter, to evaluate the cleavage efficiency of sgRNAs by measuring the expression of reporter genes. The LacI-reporter utilizes the LacI repression system, which tightly represses gene expression by binding to the *lac* operator (LacO) (41). When the synthetic target of sgRNAs, located between the EF-1α promoter and *lacI*, is cleaved via CRISPR-Cas9, the luciferase or EGFP reporter signal increases due to the lack of *lac* repression, enabling the quantification of cleavage efficiency. We experimentally quantified cleavage efficiency of sgRNAs targeting selected endogenous loci, demonstrating the reproducibility and cross-experiment comparability of this system. The LacI-reporter provides a simple and quantitative tool to select high-efficiency sgRNAs for gene editing.

## MATERIAL AND METHODS

### Construction of plasmids

CMV-lacO-Luciferase and EF-1α-LacI plasmids were provided by Peter W. Laird of the Van Andel Research Institute (41). To generate the LacI-luciferase reporter, EF-1α-multiple target sites (EcoRI, BlpI), *lacI*, and poly(A) sequences were amplified and cloned into a CMV-lacO-Luciferase vector linearized by NotI digestion using an In-Fusion HD cloning kit (TAKARA). To construct the LacI-EGFP reporter plasmid, the firefly luciferase gene in the LacI-Luciferase reporter was replaced with PCR-amplified EGFP after digestion with HindIII and XbaI. To generate the LacI-luciferase or -EGFP reporter containing the nonfunctional LacI (NF-LacI), the (helix-turn-helix HTH) DNA-binding domain was removed and cloned into LacI-luciferase or -EGFP reporter plasmids linearized by BlpI and NheI. To construct the EGFP-FKBP fusion reporter, which reduced the background expression of EGFP, the FKBP12-derived destabilizing domain (FKBP) sequence was PCR-amplified and cloned into the LacI-EGFP reporter plasmid linearized with BsrGI and PacI. To insert synthetic target sequences for guide RNAs into the LacI-reporters, one or multiple target sequences were synthesized by primer annealing or PCR amplification and inserted into the LacI-luciferase or -EGFP reporter linearized by EcoRI and BlpI. LacI-luciferase reporter (KCTC#11646) and LacI-EGFP reporter (KCTC#11647) are available in Korean Collection for Type Cultures (KCTC). For CRISPR–Cas9 constructs, we used the px459 V2.0 (Addgene #108292) vector transcribing sgRNA and transiently expressing wild-type SpCas9. Each guide sequence was subcloned into a px459 V2.0 vector linearized by BbsI. All PCR products and linearized vectors were purified with the QIAquick® Gel Extraction Kit (Qiagen). Plasmid constructs were extracted using the Plasmid DNA Miniprep S&V Kit (Bionics) and NucleoBond Xtra Midi Kit (MN) and verified by sequencing. All primers used for cloning are listed in Supplementary Table S1.

### Cell culture

HEK293T cells were maintained in Dulbecco's modified Eagle's medium (DMEM) with 10% FBS, 100 units/mL streptomycin, and 100 μg/ml penicillin. Human liver organoids were maintained in advanced Dulbecco's modified Eagle medium (ADF-12) supplemented with 1% penicillin/streptomycin, 1% GlutaMAX, 10 Mm HEPES, 10% respondin1, 25 ng/ml hHGF, 100 ng/ml hFGF10, 1X B27 supplement, 1 mM *N*-cetylcysteine, 10 nM gastrin, 10 mM nicotinamide, 1X N2 supplement, 50 ng/ml hEGF, 10 μM forskolin, and 5 μM A83-01 (42).

### LacI-luciferase reporter assay

HEK293T cells (2.5 × 10^5^ cells per well) were seeded in 12-well plates and transfected with 200 ng of LacI-luciferase or NF-LacI luciferase reporters, 100 ng of renilla, and 1800 ng of px459 (Cas9 plus or minus sgRNA) plasmid using Lipofectamine 3000 (Thermo Fisher Scientific). Cells were lysed 48 h post-transfection in passive lysis buffer (Promega) and incubated for 15 min at room temperature. After incubation, luciferase activity was measured using the Dual-Luciferase® Reporter Assay System (Promega), according to the manufacturer's instructions. In brief, 20 μl of cell lysate from 12 well plates was transferred to a 96-well white bottom plate (costar). LAR II (Luciferase Assay Buffer II + Luciferase Assay Substrate; 100 μl) was added and firefly luciferase was measured at 610 nm for 10 s using SpectraMax®Paradigm® (Molecular Devices). Luminescence was measured using a luminometer programmed to perform a 2 s premeasurement delay, followed by a 10 s measurement. Stop & Glo reagent (Stop & Glo® Substrate + Stop & Glo® reagent; 100 μl) was added and renilla was measured at the same as the firefly luciferase. For the high-throughput experiment with a 96-well plate, cells were transfected with 20 ng of LacI-luciferase reporters, 10 ng of renilla, and 180 ng of px459 (Cas9 plus or minus sgRNA) plasmid using Lipofectamine 3000 (Thermo Fisher Scientific) and lysed in 20 μl passive lysis buffer. Luciferase activity was directly measured in 96 well plates as described above. Relative luciferase activity was calculated as the ratio of firefly/renilla luciferase. The cleavage efficiencies of sgRNAs were calculated as fold change in relative luciferase activity of the experimental groups compared with those of the control groups transfected with the same plasmids without sgRNA.

### Surveyor assay

Genomic DNA was extracted using the Wizard® Genomic DNA Purification Kit (Promega), and 100 ng of was used for PCR amplification. Surveyor assays were performed using the Surveyor® Mutation Detection Kit (Integrated DNA Technologies) according to the manufacturer's instructions. In brief, PCR products were hybridized by heating and cooling the mixture to form hetero‐ and homoduplexes, then treated with surveyor nuclease at 37°C. DNA was separated by agarose gel electrophoresis and relative amounts of DNA cut by Surveyor nuclease were quantified using ImageJ software.

### Targeted deep sequencing

Genomic DNA was isolated using the Wizard® Genomic DNA Purification Kit (Promega). It was amplified by PCR using Phusion polymerase (New England Biolabs) with primers spanning the target sequence from approximately 80 bp upstream to 80 bp downstream of the cleavage site of CRISPR–Cas9. We used 100 ng of genomic DNA per sample as template for the first round of PCR amplification. PCR products were purified using a QIAquick® Gel Extraction Kit (Qiagen). For the second round of PCR, 20 ng of purified PCR products from the first round were annealed with both Illumina adapter and barcode sequences. The primers used for the PCR reactions are shown in Supplementary Table S1. The resulting products were isolated, purified, mixed, and subjected to 150 pair end sequencing using HiSeq (Illumina). Deep-sequencing data were sorted and analyzed with a reference wild-type sequence using Cas-Analyzer (43) (http://www.rgenome.net/cas-analyzer) with the comparison range (R) parameter set to 50, the minimum frequency (n) to 1, and the WT (wild-type) marker range (r) to 5. To increase the accuracy, the extracted data was filtered depending on the read number; we combined the read numbers of wild type sequences (same target sequence but in different contexts, including sequencing errors) as a single wild type sequence.

### Analysis of chromatin assessability

To identify the chromatin density level of HEK293T cells, ENCSR00EJP data (supplied by ENCODE) were used to derive the DNase-seq raw data set and DNase pipeline protocol information. SeqMonk (v.11.0.5) software was used to ensure precise chromatin accessibility analysis for accurate peak calling and log2 based quantification score calculations. A total of 23,073,839 identified peaks were divided into the following four scoring terms in a similar proportion for unbiased analysis and the peak quantification score was classified according to the degree of assembly: silent (score = 0), low level (score = 2), medium level (score = 3–4), and high level (score ≥ 5). Wherever possible, the peak classification in the human reference genome (GRCh38) was eventually determined when the number of probes and the quantitative peak score matched. For each individual peak, the proportion of each category was computed according to its length. To examine the correlation between LacI-luciferase reporter and deep sequencing, five genes (*WARS1*, *SDK1*, *CCNA1*, *GATA5*, and *BRD1*) were selected at random, and six sgRNAs were designed for each of the four categories.

### FACS analysis

HEK293T cells (2.5 × 10^5^ per well) in 12-well plates were transfected with 200 ng of LacI-EGFP reporter containing sgRNA target sequences for the *PTEN* gene, and 1800 ng of px459 (Cas9 plus sgRNA) plasmid using Lipofectamine 3000 (Thermo Fisher Scientific). Forty-eight hours after transfection, EGFP-positive cells were identified with a Navios EX Flow Cytometer (Beckman Coulter). The cleavage efficiency of sgRNAs was calculated using the EGFP mean fluorescence intensity (MFI).

### *In vitro* validation of sgRNA efficiency using a functional assay

Human liver organoids were transfected with 1800 ng of px459 plasmid (Cas9 plus sgRNA) using Lipofectamine 3000 (Thermo Fisher Scientific) as previously reported (42). In brief, single-cell suspensions at 80–90% confluency of organoids and the DNA:Lipofectamine 3000 mixture were plated and centrifuged at 32°C at 500 x *g* for one hour. Five hours after incubation, cells were seeded in 24-well plates at 80% confluency on 50 μl BME matrix. Organoids were treated with 40 μM nutlin for 6 days; then their viability was measured using a CellTiter-Glo luminescent assay based on quantitation of ATP according to the manufacturer's instructions (Promega). Luminescence was measured using a SpectraMax®Paradigm® microplate reader (Molecular Devices).

## RESULTS

### Development of a LacI-luciferase reporter to measure the cleavage efficiency of CRISPR–Cas9

**Figure 1.**
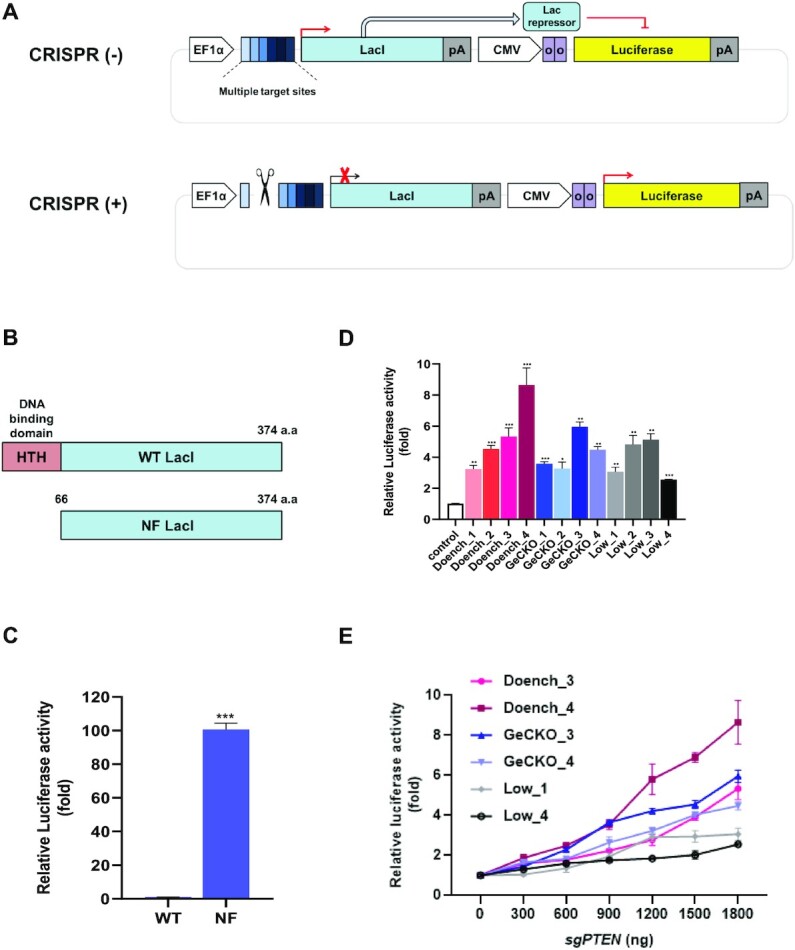
Overview of LacI-luciferase reporter and feasibility studies in human cells. (**A**) Schematic of the LacI-luciferase reporter carrying luciferase under the control of the Lac repressor. EF-1α, Human elongation factor-1α (EF-1α*)* promoter; multiple target sites, target sites for sgRNA; LacI, DNA-binding transcriptional repressor LacI; pA, polyadenylation signal; oo, double *lac* operator; CMV, cytomegalovirus promoter. (**B**) Schematic of nonfunctional LacI showing the truncation of the HTH (helix-turn-helix) DNA-binding domain. (**C**) Luciferase activity regulated by the *lac* repressor in the LacI-luciferase reporter. Wild-type and nonfunctional LacI were transfected into HEK293T cells. The wild-type *lac* repressor, not the nonfunctional *lac* repressor, repressed luciferase activity in the LacI-luciferase reporter (*n* = 3; mean ± SD; ***P < 0.001; unpaired *t*-test). (**D**) Feasibility of LacI-luciferase reporter for assessing the cleavage efficiency of sgRNA. Twelve sgRNAs targeting the *PTEN* gene were designed based on previous studies (13,15,16,38,39), denoted the Doench, GeCKO, and Low groups. The px459 plasmid expressing sgRNA and Cas9, the LacI-luciferase reporter carrying target sites for 12 sgRNAs, and renilla were co-transfected into HEK293T cells. Luciferase activity was normalized to renilla and fold change was calculated relative to the control group, which was transfected with the same plasmids minus sgRNA (*n* = 3; mean ± SD). (**E**) The luciferase activity of the reporter depends on CRISPR–Cas9 cleavage efficiency. HEK293T cells were co-transfected with different concentrations of sgRNAs targeting *PTEN* selected from (**D**) and the LacI-luciferase reporter. (*n* = 3; mean ± SD).

We exploited the LacI repression system, in which the *lac* repressor suppresses gene expression by binding to the *lac* operator (41,44). We developed a LacI-luciferase reporter consisting of a *lac* repressor under control of the EF-1α promoter, as well as a luciferase reporter under the control of the CMV promoter containing the *lac* operator. The *lac* repressor binds to the *lac* operator located between the CMV promoter and the luciferase reporter, repressing the expression of the luciferase reporter. Of note, when CRISPR–Cas9 cleaves targets located between the EF-1α promoter and the *lacI* gene, *lacI* cannot be transcribed, leading to the induction of luciferase expression. We hypothesized that the cleavage efficiency of CRISPR–Cas9 could be quantified by measuring the luciferase reporter activity (Figure 1A). To examine whether luciferase activity can be regulated by the *lac* repressor, nonfunctional LacI (NF-LacI) was produced by truncating the helix-turn-helix (HTH) of the *lac* repressor, which is required for binding to the *lac* operator (Figure 1B). In the NF-LacI group, the activity of luciferase increased by 100-fold over that of the wild-type *lac* repressor, indicating that the *lac* repressor regulates luciferase reporter activity (Figure 1C). To examine whether the LacI-luciferase reporter can measure differences in cleavage efficiencies of sgRNAs, we designed 12 sgRNAs for the *PTEN* gene that cover a wide range of sgRNA efficiencies based on previous studies. We chose the four highest scoring sgRNAs designed using the CRISPRko (20,21) webtool, four sgRNAs from the GeCKOv2 (22,45) libraries, and four sgRNAs that were expected to be inefficient based on their GC content (32), designated as the Doench, GeCKO, and Low groups, respectively. The cleavage efficiencies of these twelve sgRNAs were evaluated by measuring the relative luciferase activity, which ranged from 2- to 8-fold higher than that of the control group (Figure 1D). This experiment was scaled to 96-well plates and reproduced, suggesting its usability for high-throughput screening (Supplementary Figure S1). Among the twelve sgRNAs, we selected six, two each from the Doench, GeCKO, and Low groups according to the range of cleavage efficiencies, and examined whether LacI-luciferase activity reflected sgRNA-Cas9 cleavage efficiency (Figure 1E). Luciferase activity depended on the concentration of sgRNA-Cas9, indicating that it can be used as an estimate of cleavage efficiency. We chose the 1:9 ratio of the LacI-luciferase reporter to the CRISPR–Cas9 plasmid to measure cleavage efficiency by CRISPR-Cas9 in subsequent experiments. Furthermore, we examined whether blocking the religation of the LacI-luciferase reporter after cutting by CRISPR–Cas9 improved its sensitivity. Treatment with SCR7, an NHEJ inhibitor, did not affect the efficiency of the LacI-reporter (Supplementary Figure S2).

### Comparison of LacI-luciferase activity and indel frequency

**Figure 2.**
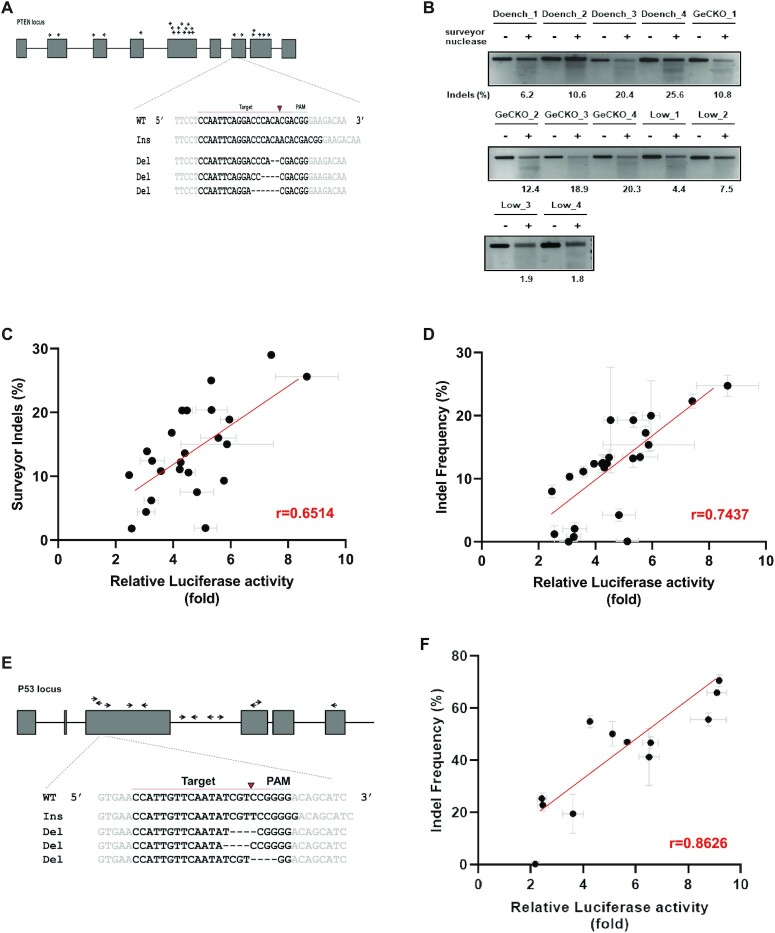
Assessment of the cleavage efficiency of sgRNAs using the LacI-luciferase reporter. (**A**) Schematic of sgRNAs targeting the *PTEN* gene and representative deep sequencing data. The 12 sgRNAs for *PTEN* were designed to determine their cleavage efficiency using the LacI-luciferase reporter and indels using deep sequencing. Representative views of indels at the indicated loci are shown. Red arrowheads denote predicted Cas9 cutting sites. Black lines denote deletions and red letters denote insertions. (**B**) Representative images of the surveyor assay of 12 sgRNAs for *PTEN*. Indel frequencies of 24 sgRNAs were analyzed using a surveyor assay. The indel ratios were calculated based on band intensities. (**C**) Correlation between the LacI-luciferase reporter and surveyor assay data. The cleavage efficiency of 24 sgRNAs was measured using the LacI-luciferase reporter and the indel frequency was assessed using a surveyor assay (**B**). Correlation was calculated using Pearson's correlation coefficient, *r* = 0.6514, (*n* = 3) (**D**) Correlation between the LacI-luciferase reporter and deep sequencing data. For 24 sgRNAs, the cleavage efficiency was assessed using the LacI-luciferase reporter assay and the indel frequency was analyzed using deep sequencing. The correlation was calculated using Pearson's correlation coefficient, *r* = 0.7437, (*n* = 3; mean ± SD). (**E**) Schematic of sgRNAs targeting the *TP53* gene and representative deep sequencing data. Twelve sgRNAs for the *TP53* gene were designed to examine cleavage efficiency using the LacI-luciferase reporter assay and the indel frequency using deep sequencing. Representative views of indels at the indicated loci are shown. Red arrowheads denote the predicted Cas9 cutting sites. Black lines denote deletions and red letters denote insertions. (**F**) Correlation between the results of the LacI-luciferase assay and deep sequencing. For 12 sgRNAs, the cleavage efficiency was assessed using the LacI-luciferase reporter and the indel frequency was analyzed using deep sequencing. The correlation between the two methods was calculated using Pearson's correlation coefficient, *r* = 0.8626, (*n* = 3; mean ± SD).

To examine whether the cleavage efficiency of sgRNAs determined using the LacI-luciferase reporter is concordant with other assays, we compared results from the LacI-luciferase reporter with those of the surveyor assay and deep sequencing. We designed 24 sgRNAs targeting the *PTEN* gene (Figure 2A) and inserted their sequences into the LacI-luciferase reporter. We evaluated whether the cleavage efficiency at the synthetic target in the LacI-reporter plasmid correlated with the indel frequency at the corresponding endogenous target. Surveyor analysis was performed on 24 sgRNAs (Figure 2B); we observed a correlation (*r* = 0.6514) between the LacI-luciferase reporter and the surveyor assay (Figure 2C). We analyzed the indel frequency by deep sequencing at the endogenous target for 24 sgRNAs and observed a correlation (*r* = 0.7437) between the LacI-luciferase reporter and deep sequencing results. To examine whether this correlation is gene-specific, we designed 12 sgRNAs for the *TP53* gene (Figure 2E). Similarly, we observed a strong correlation (*r* = 0.826) between the LacI-luciferase reporter and deep sequencing results for TP53 (Figure 2F). These results indicate that the LacI-luciferase reporter can quantitatively measure the cleavage efficiency of sgRNA in different genes similarly to the surveyor assay and deep sequencing.

### Effect of length and arrangement of sgRNA targets on the LacI-luciferase reporter

**Figure 3.**
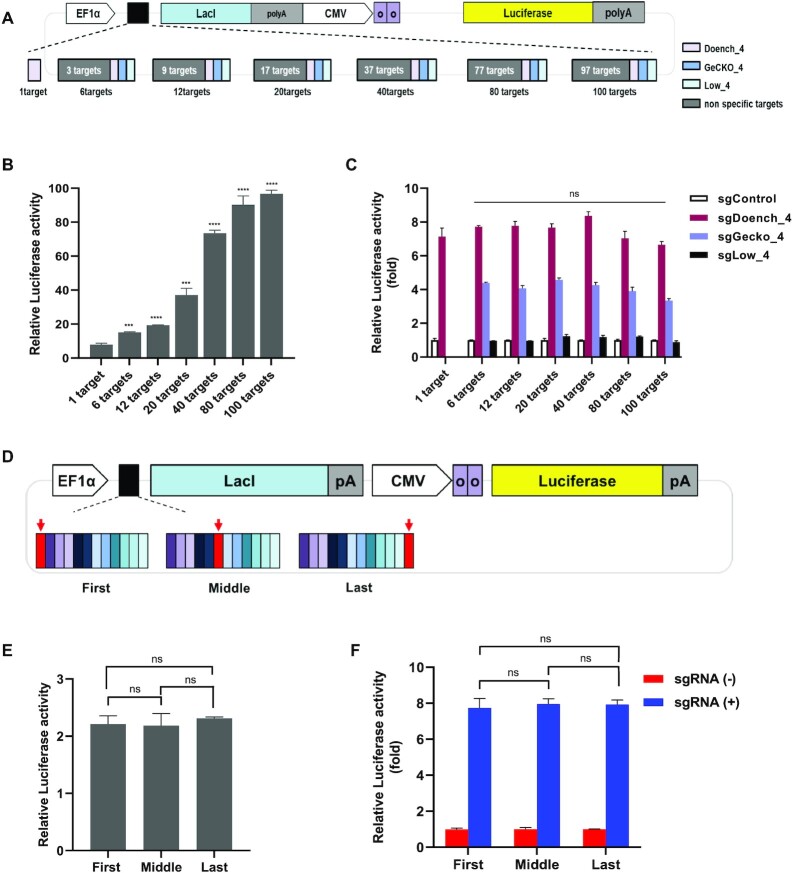
Effects of length and arrangement of synthetic sgRNA targets in the LacI-luciferase reporter. (**A**) Schematic showing lengths of sgRNA target sequences between the EF-1α promoter and LacI. (**B**) Effect of sgRNA target sequence length. Without cutting the LacI-luciferase reporter, the LacI-luciferase reporter carrying different numbers of sgRNA targets, Cas9, and renilla were cotransfected into HEK293T cells. Reporter luciferase activity was normalized to renilla (*n* = 3; mean ± SD, ***P < 0.001; unpaired *t*-test). (**C**) LacI-luciferase reporter carrying different numbers of sgRNA targets, Cas9 plus sgRNA, and renilla were co-transfected into HEK293T cells. Reporter luciferase activity was normalized to renilla and fold change was calculated relative to the control group, which was transfected without sgRNA. The length of sgRNA target sequence did not affect the reporter luciferase activity (*n* = 3; mean ± SD, ns = not significant, unpaired *t*-test). (**D**) Schematic showing arrangements of target sequences between the EF-1α promoter and LacI. The sgRNA target sequence was placed in the first, middle, or last position in multiple target sites. (**E**) Without cutting the LacI-luciferase reporter, the LacI-luciferase reporter with different arrangements of sgRNA targets, Cas9, and renilla were co-transfected into HEK293T cells. The reporter luciferase activity was normalized to renilla. The position of the target sequence did not affect the reporter luciferase activity (*n* = 3; mean ± SD, ns = not significant, unpaired *t*-test). (**F**) LacI-luciferase reporter carrying different numbers of sgRNA targets, Cas9 plus sgRNA, and renilla were co-transfected into HEK293T cells. The reporter luciferase activity was normalized to renilla and fold change was calculated relative to the control group, which was transfected without sgRNA. (*n* = 3; mean ± SD, ns = not significant, unpaired *t-*test).

The length and/or arrangement of synthetic target sequences in the LacI-luciferase reporter may affect the expression of the lac repressor, thereby influencing the luciferase activity, which reflects sgRNA cleavage efficiency. First, we examined the effect of the length of sgRNA target sequences on the LacI-luciferase reporter (Figure 3A). When the LacI-luciferase reporter and Cas9 were present without sgRNA, as the number of sgRNA targets increased luciferase activity increased (Figure 3B). It is assumed that longer distances between the EF-1α promoter and *lacI* decrease LacI expression, which affects luciferase activity. However, when the cleavage efficiency of sgRNA measured using the LacI-luciferase reporter was normalized to the corresponding LacI-luciferase reporter control that was not cut by sgRNA, the effect of the length of sgRNA target sequence on the LacI-luciferase reporter was eliminated (Figure 3C). Therefore, the cleavage efficiency of sgRNAs measured between LacI-luciferase reporters can be compared despite the LacI-luciferase reporters containing different numbers of sgRNA targets. Second, we examined the position effect of an sgRNA target in a LacI-luciferase reporter. To examine this, we measured the cleavage efficiency of sgRNA using reporters that have the same number of sgRNA targets but have different arrays, in which an sgRNA target is located in the first, middle, or last of 12 sgRNA targets (Figure 3D). In addition, a common 20-bp sequence (M13 forward) was inserted directly in front of the *lacI* gene to prevent the effect of the target sequence at the last position from affecting its transcription or translation. We found that there was no significant difference among LacI-luciferase reporters that had different arrays of an sgRNA target without cutting by CRISPR-Cas9 (Figure 3E). When the cleavage efficiency of sgRNA measured using the LacI-luciferase reporter was normalized to the corresponding LacI-luciferase reporter control that was not cut by sgRNA, there was no position effect of sgRNA (Figure 3F). Together, the LacI-luciferase reporter system can measure the cleavage efficiency of sgRNAs regardless of the lengths and arrangements of sgRNA targets.

### Effect of chromatin status on the LacI-luciferase reporter assay

**Figure 4.**
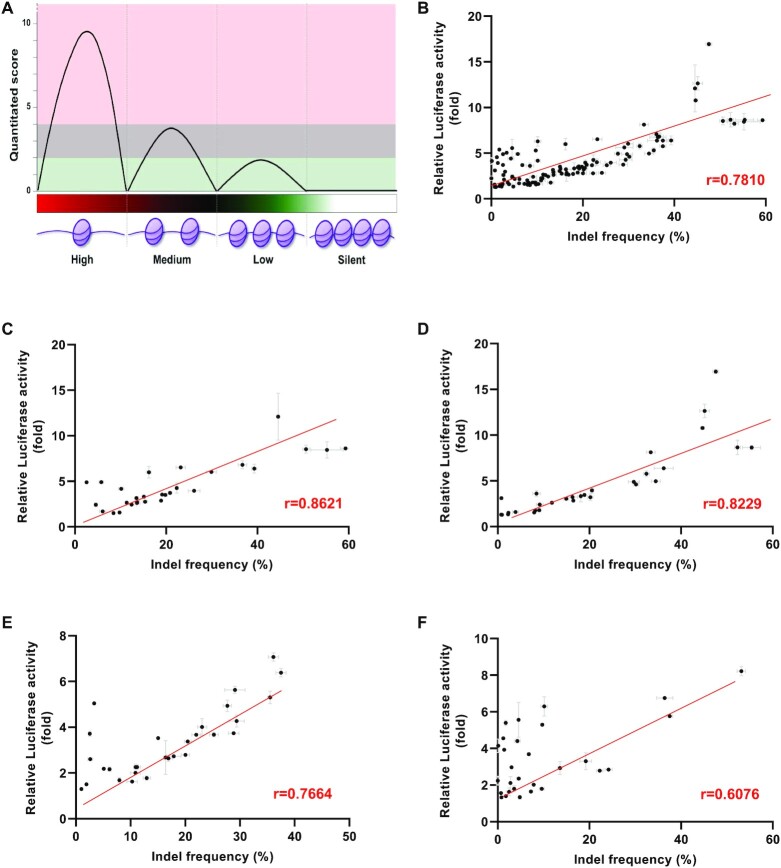
Effect of chromatin status on the LacI-luciferase reporter assay. (**A**) Conceptual design of Chromatin assessability. The computed peak with the four scoring terms based on peak calling results and peak quantitative score. Each scoring term provides information on a total peak score and number of probes. High level, score ≥5; medium level, score = 3–4; low level, score = 2; silent, score = 0. Within five randomly selected genes, the six single guide RNAs were designed for each of the four groups. Correlations between cleavage efficiencies according to the LacI-luciferase reporter and indel frequencies measured using deep sequencing were determined for a total of 118 sgRNAs targeting endogenous sites covering all four groups (high, medium, low, and silent) (**B**), high group only (**C**), medium group only (**D**), low group only (**E**), and silent group only (F).

Chromatin accessibility can affect the efficiency of CRISPR–Cas9-mediated gene editing as closed chromatin inhibits on-target Cas9/sgRNA–DNA binding (46–50). To examine whether the LacI-luciferase reporter can measure the cleavage efficiency of sgRNA at endogenous target sites with different chromatin accessibilities, we divided DNase I hypersensitivity into four groups (high, medium, low, and silent) in Figure 4A, according to the level of chromatin accessibility in the whole genome (Supplementary Figure S3). ENCSR00EJP for DNase-seq raw dataset (51) and DNase pipeline information were used to identify chromatin density levels in HEK293T cells. To classify peak quantitation score according to its assembling degree, a total of 23,073,839 peaks were sorted into four scoring terms as follows: silent (score = 0), low (score = 2), medium (score = 3–4), and high (score ≥ 5) (Figure 4A). To examine the correlation between LacI-luciferase reporter and deep sequencing, five genes (*WARS1*, *SDK1*, *CCNA1*, *GATA5* and *BRD1*) located in the different chromosomes were randomly selected, and six sgRNAs were designed in each of the four groups (Supplementary Figure S4). The correlation between LacI-luciferase and deep sequencing results for a total of 118 sites (two sites were failed for amplification) was *r* = 0.7810 (Figure 4B). When 118 target sites were divided into four groups, the correlation between the LacI-luciferase reporter and deep sequencing results was *r* = 0.8821 (high), *r* = 0.8229 (medium), *r* = 0.7664 (low) and *r* = 0.6076 (silent), respectively (Figure 4C–F). Although the correlation between LacI-luciferase and deep sequencing was slightly lower in the silent group with no chromatin accessibility, the correlation was still high in all four groups. Interestingly, the correlation between cleaving frequency from the CRISPRko webtool and deep sequencing was lower than that between LacI-luciferase and deep sequencing, suggesting that the LacI-luciferase reporter could compensate for the gRNA design tool to select the efficient gRNA (Supplementary Figure S5).

### Development of a LacI-EGFP reporter to measure the cleavage efficiency of CRISPR–Cas9

**Figure 5.**
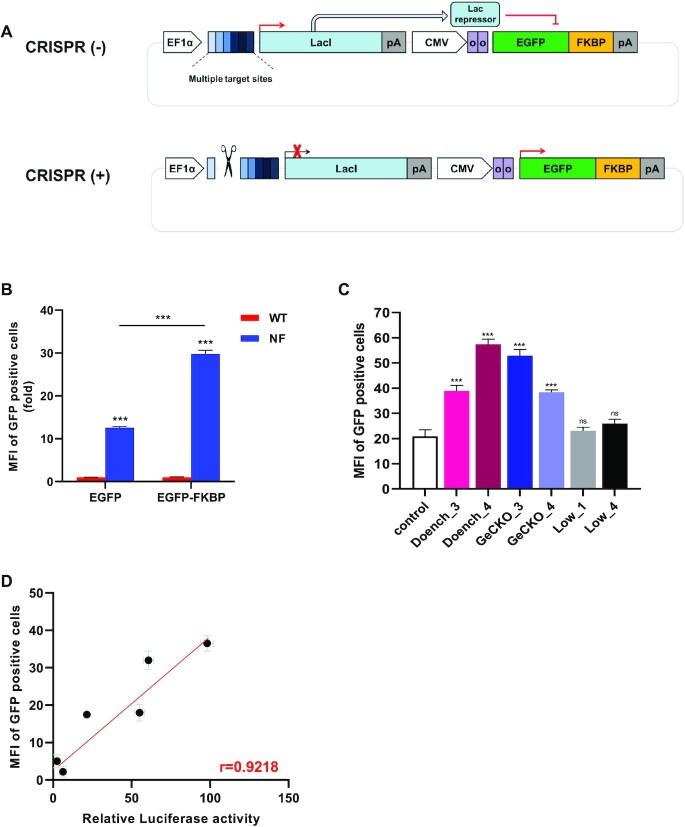
Overview of the LacI-EGFP reporter and feasibility studies in human cells. (**A**) Schematic of the LacI-EGFP reporter carrying EGFP-FKBP regulated by Lac repressor. EF-1α, Human elongation factor-1α (EF-1α) promoter; multiple target sites, target sites for sgRNA; LacI, DNA-binding transcriptional repressor LacI; pA, polyadenylation signal sequence; oo, double lac operator; CMV, cytomegalovirus promoter; FKBP, FKBP12-derived destabilizing domain. (**B**) Enhancement of signal-to-noise ratio of EGFP with EGFP-FKBP. MFI (mean fluorescence intensity) of EGFP was measured and fold change was calculated as the MFI of the non-functional Lac repressor over that of the wild type repressor (*n* = 3; mean ± SD; ***P < 0.001; unpaired *t*-test). EGFP-FKBP reduced EGFP background and increase EGFP signal compared to EGFP in the LacI-EGFP reporter. (**C**) Feasibility of using the LacI-EGFP reporter to measure the cleavage efficiency of sgRNAs. The px459 (sgRNA plus Cas9) and LacI-EGFP reporter plasmids were transfected into HEK293T cells. The MFI of EGFP was measured and the fold change was calculated as the MFI of the experimental groups divided by that of the control. (**D**) Correlation of the cleavage efficiency of sgRNAs measured using the LacI-luciferase reporter and LacI-EGFP reporter assays. Pearson correlation coefficient *r* = 0.9218, *P* = 0.0070, (*n* = 3; mean ± SD).

We developed a reporter system to assess the cleavage efficiency of sgRNA by measuring EGFP expression by replacing luciferase with EGFP in the LacI-luciferase reporter (Figure 5A). To determine whether EGFP expression can be regulated by the lac repressor, nonfunctional *lacI* (NF-LacI) was produced by truncating the helix-turn-helix (HTH) of the *lac* repressor, similar to the NF-LacI-luciferase reporter (Figure 5B). As expected, EGFP expression in the nonfunctional LacI (NF-LacI) control was significantly higher than in wild-type LacI. The LacI-EGFP reporter system had a high background of EGFP expression, resulting in lower signal sensitivity. To reduce background expression of EGFP, FKBP (FKBP12-derived destabilizing domain), a destabilizing domain, was fused to the C-terminus of EGFP (Figure 5B and Supplementary Figure S6A). We chose 6 sgRNAs for the *PTEN* gene that were tested for the LacI-luciferase reporter to assess cleavage efficiency using the LacI-EGFP reporter (Figure 5C and Supplementary Figure S6B). We compared the cleavage efficiency of sgRNAs measured using the LacI-EGFP reporter and the LacI-luciferase reporter, and found that the correlation between the two different LacI-reporter results was *r* = 0.9218. This indicates that the efficiency of sgRNA can be quantitatively measured using both the LacI-luciferase reporter and the LacI-EGFP reporter. We tested the normalization of the LacI-EGFP reporter to mcherry in one- and two-vector systems (Supplementary Figure S7A). The expression of mcherry decreased according to the activity of gRNA in the one-vector system, whereas there was no difference in mcherry expression in the two-vector systems that separately expressed the mcherry and LacI-EGFP reporter (Supplementary Figure S7B, C, and D). We also tested different concentration of LacI-EGFP reporter and mcherry and found a minor increase in reporter activity compared with that using the mcherry control (Supplementary Figure S7E). We also found similar results for LacI-luciferase and renilla control (Supplementary Figure S7F). This data shows that variation from transfection efficiency has little effect on the activity of the reporter, since the LacI-reporter is silenced without cleavage by CRISPR-Cas9. Thus, if there is variation, it is likely to be from the transfection of CRISPR-Cas9. The average percentage of the standard error mean of LacI-reporter and corresponding deep sequencing results from 446 data points were 3.76% and 7.6%, respectively, indicating that the variation of our reporter was no higher than that of deep sequencing results, and therefore, both methods would be similarly affected by transfection of CRISPR-Cas9.

### Validation of sgRNA efficiency in human cells

**Figure 6.**
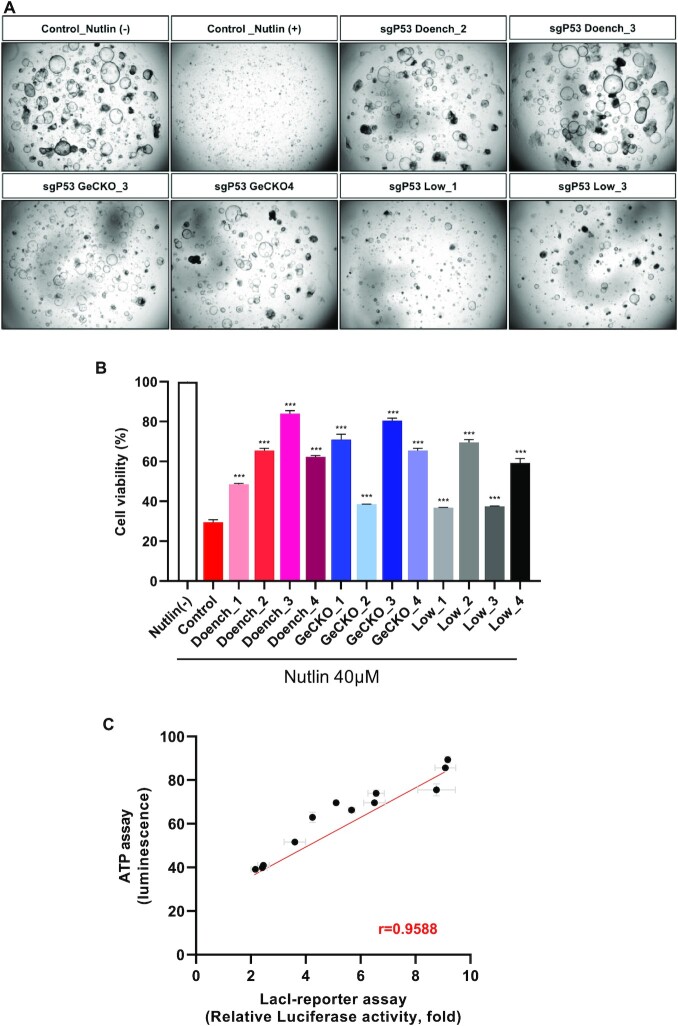
Evaluation of sgRNA efficiency in human liver organoids. (**A**) Representative images following the introduction of *TP53* sgRNAs. Human liver organoids were transfected with CRISPR–Cas9 and *TP53* sgRNAs, then selected with nutlin-3, an mdm2 inhibitor, for 72 h. (**B**) Cell viability of human liver organoids transfected with CRISPR–Cas9 for the *TP53* gene. Cell viability (ATP content) was measured at 72 h after nutlin-3 selection. (**C**) Correlation between the cleavage efficiencies of sgRNAs measured using the LacI-luciferase reporter and viabilities measured using the ATP assay. Pearson correlation coefficient *r* = 0.9588, *P* = 0.0014, (*n* = 3; mean ± SD).

To determine whether the cleavage efficiency of sgRNAs measured by the LacI-luciferase reporter was maintained in human organoid cultures, we selected six sgRNAs for the *TP53* gene for which the cleavage efficiency had been measured using the LacI-luciferase reporter, as shown in Figure 2F. To select *TP53* deficient organoids by CRISPR–Cas9, nutlin selection was performed for 72 h in human liver organoids. Nutlin, an MDM2 inhibitor, inhibits the interaction between MDM2 and TP53, resulting in TP53 stabilization and apoptosis, whereas there is no effect in TP53-deficient cells (52,53). Nutlin treatment completely killed organoids in the control group, but the survival rate of sgRNA/Cas9 transfected organoids varied depending on the cleavage efficiency of the sgRNAs (Figure 6A). Cell viability was measured by ATP assay at 72 h after treatment with 40 μM nutlin (Figure 6B). There was a correlation between viability and sgRNA cleavage efficiency measured using the LacI-luciferase reporter (*r* = 0.97), indicating that efficient sgRNAs can be identified using this system for gene editing (Figure 6C).

## DISCUSSION

We developed a novel cleavage-based surrogate reporter to measure the cleavage efficiency of sgRNAs based on the LacI repression system. The LacI repressor tightly represses reporter expression by binding to the *lac* operator. When CRISPR–Cas9 cuts target sequences in the LacI-reporter, LacI cannot be transcribed due to the lack of its promoter, inducing the expression of the reporter gene. In the present study, we developed two reporters, LacI-luciferase and LacI-EGFP, and used them to evaluate the cleavage efficiency of sgRNAs. We then compared our LacI-reporters with other common methods to determine sgRNA efficiency. We also validated the gene-editing efficiency of sgRNAs in human liver organoids, demonstrating successful measurement of the cleavage efficiency of sgRNAs using the LacI-reporter.

The LacI-reporter is a quantitative method for the evaluation of the cleavage efficiency of sgRNAs. PCR-based methods, including Surveyor, T7E1 assay, and targeted deep sequencing, have the potential to generate errors because of their dependence on DNA polymerase. The Surveyor and T7E1 assays can also produce false-positive results, as heteroduplexes caused by polymorphisms in the genome can be recognized as mutations by CRISPR–Cas9. In addition, a single LacI-reporter can contain up to 120 sgRNAs, which makes it possible to select efficient gRNA with high throughput for one or several genes, thus, saving time and cost. Most surrogate reporter systems evaluate targeted mutagenesis, the consequence of DNA repairs after being cleaved by CRISPR-Cas9, by measuring frameshift mutations of the reporter gene. As a result, in-frame mutations that do not affect the expression of reporters, such as insertions or deletions of 3n base pairs by CRISPR–Cas9, can be overlooked. In contrast, the LacI-reporters can directly measure the cleavage by CRISPR-Cas9, which is independent of DNA repair systems, by measuring the reporters that are expressed only in the cleaved LacI-reporter, not uncleaved or repaired LacI-reporter by DNA repair systems. Another surrogate reporter, universal donor as reporter (UDAR), evaluates the efficiency of CRISPR–Cas9 by measuring the expression of EGFP that is integrated into the DSB region of the target gene using the NHEJ-mediated knock-in strategy (12,14,34). However, a homology-independent knock-in strategy may cause nonspecific integration of the EGFP donor by off-target effects of CRISPR–Cas9 (14). Since the UDAR reporter system uses a poly(A)-free EGFP donor, an endogenous polyA signal is required for the expression of the integrated EGFP, limiting its use to genes that are transcribed with a poly(A) signal. The LacI-reporter measures the cleavage efficiency of sgRNA for the synthetic target sequence, thus excluding the off-target effects of CRISPR–Cas9. The LacI-reporter is a more accurate system for the determination of cleavage efficiency of sgRNAs because it avoids the measurement of CRISPR–Cas9 cleavage and DNA repair processes.

Although gRNA design algorithms have been improved and are widely used to select candidate guide RNAs for target genes, T7E1, surveyor assay, and targeted deep sequencing are commonly used to experimentally validate the efficiency of candidate guide RNAs; however, all of these methods require genomic amplification of each target region corresponding to the gRNA, which is inefficient in measuring the efficiency of sgRNA for multiple target sites. In contrast, one LacI-reporter plasmid can contain up to 120 gRNA target sites for one or multiple genes and, thus, can be used to compare the efficiency of candidate gRNAs with comparable accuracy to surveyor assay and deep sequencing. The LacI-reporter was optimized to simultaneously measure the efficiency of multiple sgRNAs. We obtained consistent results using the LacI-reporter with different numbers and arrangements of target sequences. The length effect caused by different numbers of targets was eliminated by normalization of sgRNA activity to the non-cleavage control. In addition, we demonstrated that target-sequence arrangement does not affect the performance of our reporter system, indicating that multiple sgRNAs can be compared simultaneously, thus improving throughput.

Since the LacI-reporter is an episomal surrogate system, the assay cannot account for the difference in chromatin state. Nevertheless, the correlation between the LacI-luciferase reporter and deep sequencing results was high in the low, medium, and high chromatin accessibility regions but was slightly lower in the silent chromatin accessibility region, which is concordant with the results of previous studies. Chung et al. reported the required level of DNA accessibility for CRISPR–Cas9 reaction is significantly less than that used for endogenous genes to be expressed (54). In this regard, this system is still useful for selecting the most efficient gene-editing sgRNA, as a large number of DNase I-sensitive regions closely approximated or overlapped with annotated exons (55). We demonstrated LacI-reporter usability for multiple sgRNAs in *PTEN* and *TP53* genes and validated the LacI-reporter using the *TP53* gene in human hepatic organoids. We expect that the LacI-reporter could be applied to other genes and intend to validate this system in the context of different cell types. In conclusion, the LacI-reporter developed in the present study is a novel cleavage-based surrogate reporter to evaluate the efficiency of sgRNA cleavage simply and accurately, enabling the selection of efficient sgRNAs for genome editing.

## Supplementary Material

gkab467_Supplemental_Files
