# Supplementary material for: A cleavage-based surrogate reporter for the evaluation of CRISPR–Cas9 cleavage efficiency
Source: Nucleic Acids Res. 2021 Jun 4;49(15):e85. doi: 10.1093/nar/gkab467 (PMC8421217; doi:10.1093/nar/gkab467)
Supplement: gkab467_Supplemental_Files [file gkab467_supplemental_files.zip › Jung_et_al_Supplemental Figure legends.docx]

SUPPLEMENTAL FIGURE LEGENDS

Supplemental Figure 1. Feasibility of a LacI-luciferase reporter for assessing the cleavage efficiency of sgRNA in 96 well plates. LacI-luciferase reporter carrying target sites for 12 sgRNAs used in Figure 1D, and renilla were co-transfected into HEK293T cells. Luciferase activity was normalized to renilla and fold change was calculated relative to the control group, which was transfected with the same plasmids minus sgRNA (*n* = 3; mean ± SD).

Supplemental Figure 2. Effect of NHEJ inhibition on the LacI-luciferase reporter. SCR7, an NHEJ inhibitor, did not affect luciferase activity in the LacI-luciferase reporter with either wild type and nonfunctional LacI (A), or the cleavage efficiency of different sgRNAs in HEK293T cells (*n =* 3; mean ± SD) (B).

Supplemental Figure 3. Distribution of DNase I hypersensitive sites in the whole genome with the four scoring terms according to peak quantitative score.

Supplemental Figure 4. Schematic of sgRNAs targeting *WARS2*, *SDK1*, *CCNA1*, *GATA5*, and *BRD1*. Within five randomly selected genes, six single guide RNAs were designed for each of the four categories. Colors from red to white represents peak quantitation score according to probe of assemblage for DNase I accessibility. Red (high), orange (medium), yellow (low), green (silent) arrowheads denote predicted Cas9 cleavage sites by designed sgRNAs.

Supplemental Figure 5. Correlations between deep sequencing results and cleaving frequency predicted by the CRISPRko webtool. Correlations between cleavage efficiencies measured using the LacI-luciferase reporter and cleaving frequency predicted by the CRISPRko webtool, for a total of 118 sgRNA target sites covering all four groups (high, medium, low, and silent) (A), high group only (B), medium group only (C), low group only (D), and silent group only (E).

Supplemental Figure 6. FACS scatter plots for the LacI-EGFP reporter shown in Figure 5. (A) FACS scatter plots for HEK293T, LacI-EGFP, LacI-EGFP–FKBP, and NF-LacI-EGFP group shown in Figure 5B. (B) FACS scatter plots for six different sgRNAs shown in Figure 5C. The px459 (sgRNA plus Cas9) and LacI-EGFP reporter plasmids were transfected into HEK293T cells. MFI (mean fluorescence intensity) of EGFP was shown in red.

Supplemental Figure 7. Effect of LacI-reporter on experimental variation. (A) Schematic of the one- and two-vector systems of the LacI-EGFP reporter. One-vector system consists of LacI-EGFP reporter and mcherry under control of the PGK promoter on the same plasmid. The two-vector system involves separate expression of the LacI-EGFP reporter and mcherry. (B) In the one-vector system, the expression of mcherry decreased according to the activity of gRNA, which may be attributable that the linearized DNA that is generated by CRISPR/Cas9 is more susceptible to degradation than uncleaved circular DNA. (C) In contrast, there was no difference in mcherry expression in the two-vector systems. (D) MFI (mean fluorescence intensity) of EGFP in LacI-EGFP reporter was normalized to MFI of mcherry as the activity of firefly luciferase in LacI-luciferase reporter was normalized to renilla in one-vector and two-vector systems. (E) MFI of LacI-EGFP and mcherry transfected with different concentrations of DNA. (F) Luciferase activity of LacI-luciferase reporter and renilla transfected with different concentrations of DNA.
