# Supplementary material for: A cleavage-based surrogate reporter for the evaluation of CRISPR–Cas9 cleavage efficiency
Source: Nucleic Acids Res. 2021 Jun 4;49(15):e85. doi: 10.1093/nar/gkab467 (PMC8421217; doi:10.1093/nar/gkab467)

Supplemental Figure 1

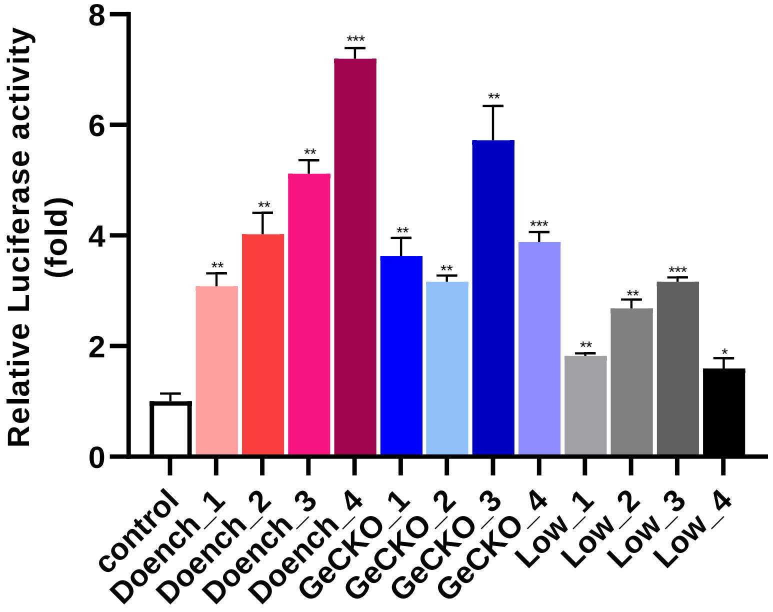

Supplemental Figure 2

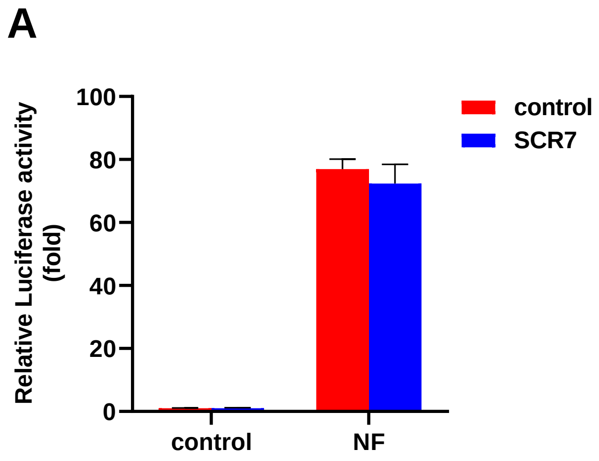

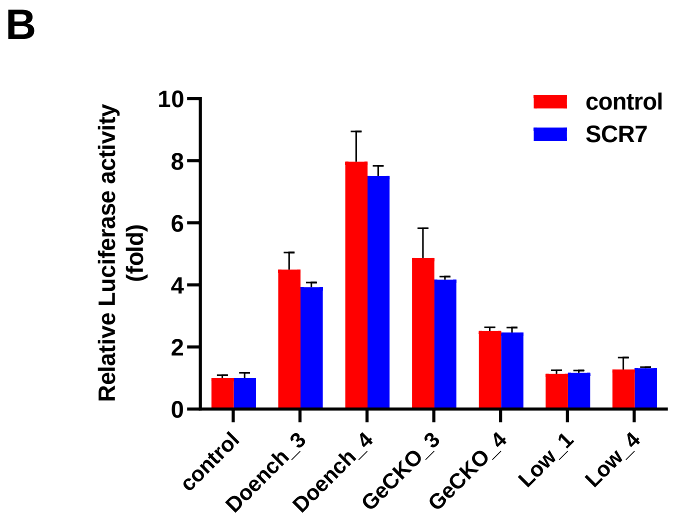

Supplemental Figure 3

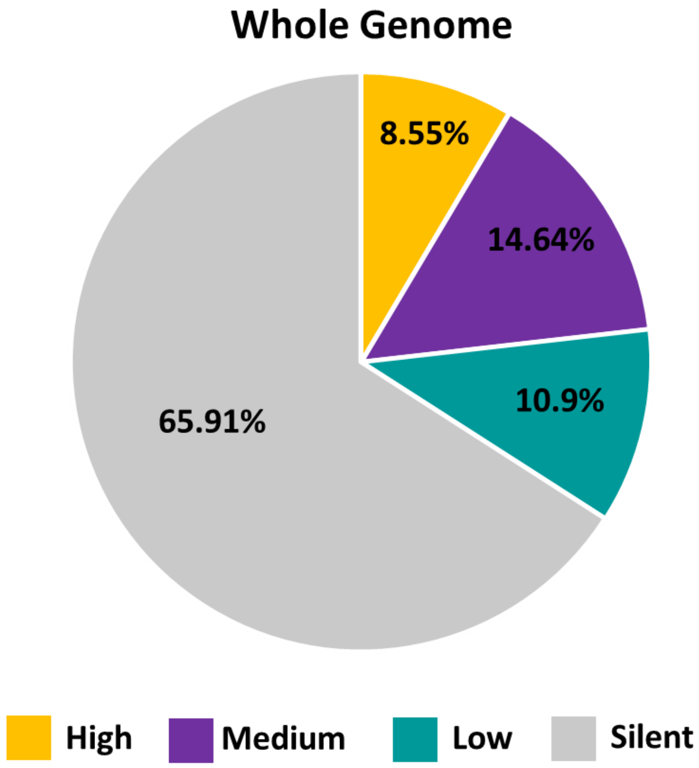

Supplemental Figure 4

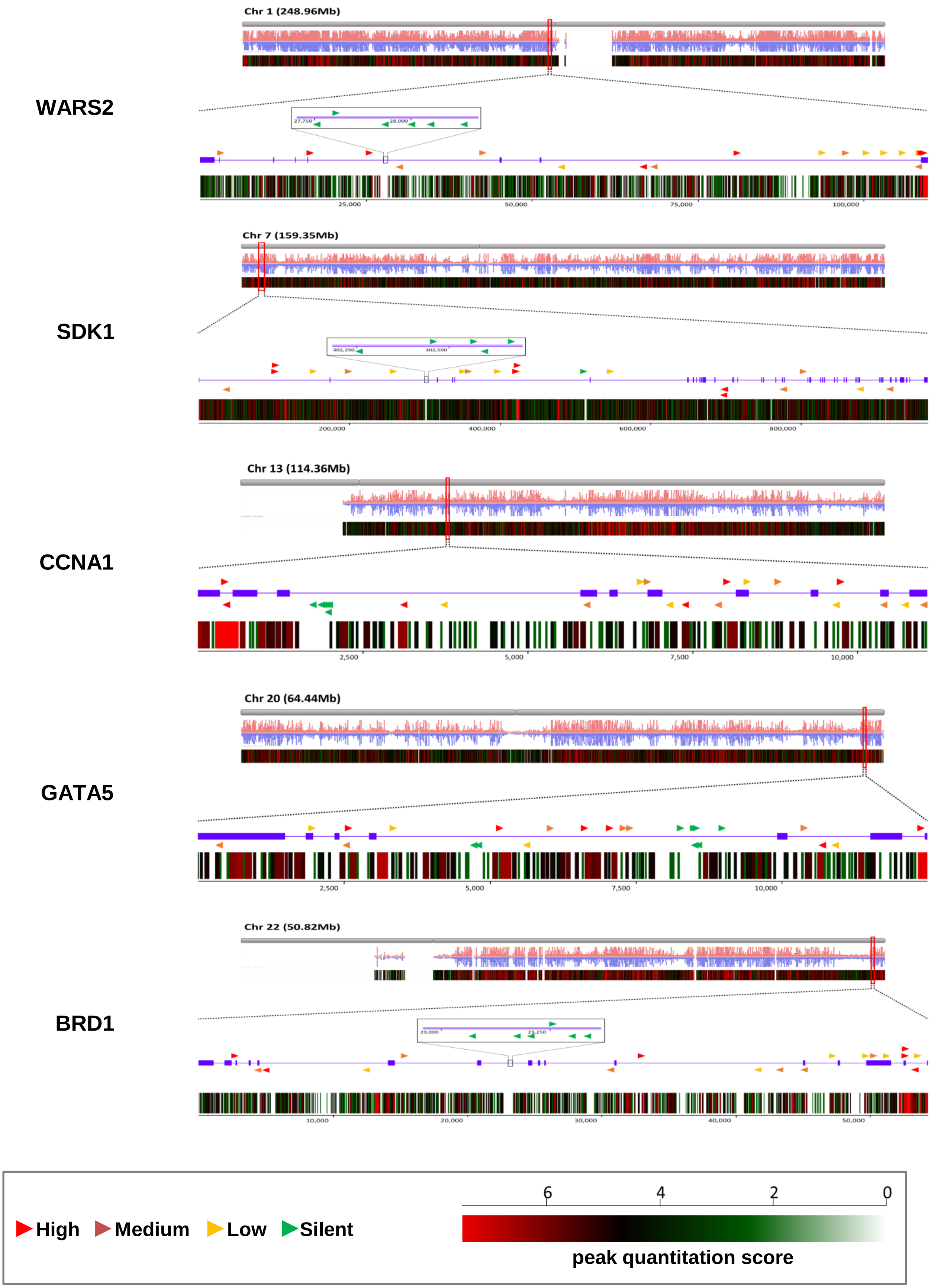

Supplemental Figure 5

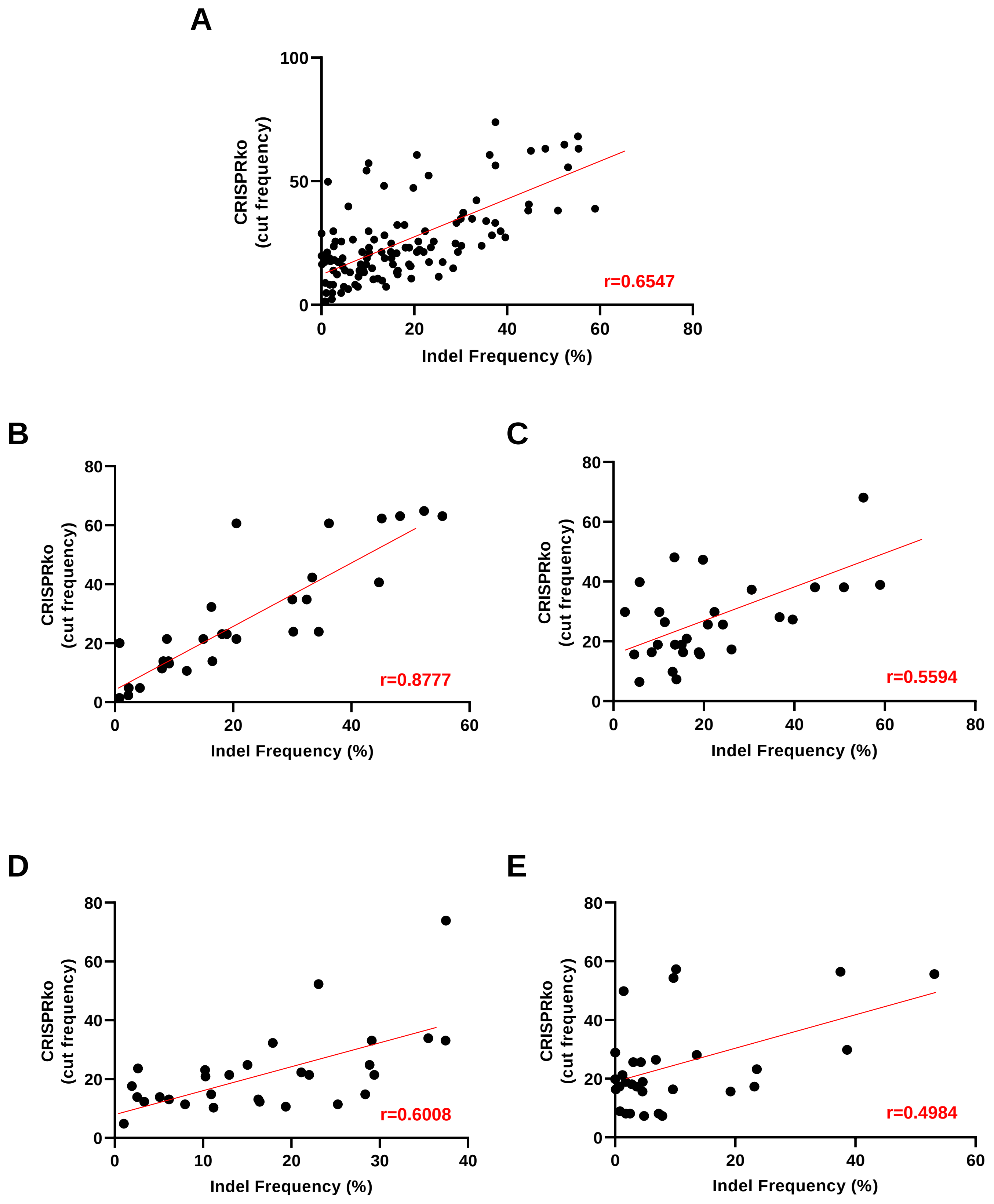

Supplemental Figure 6

A

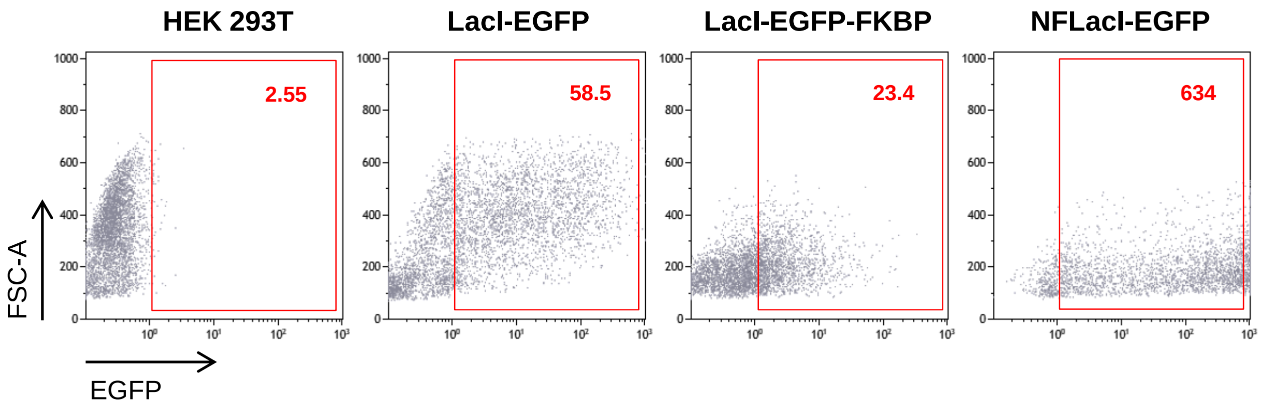

B

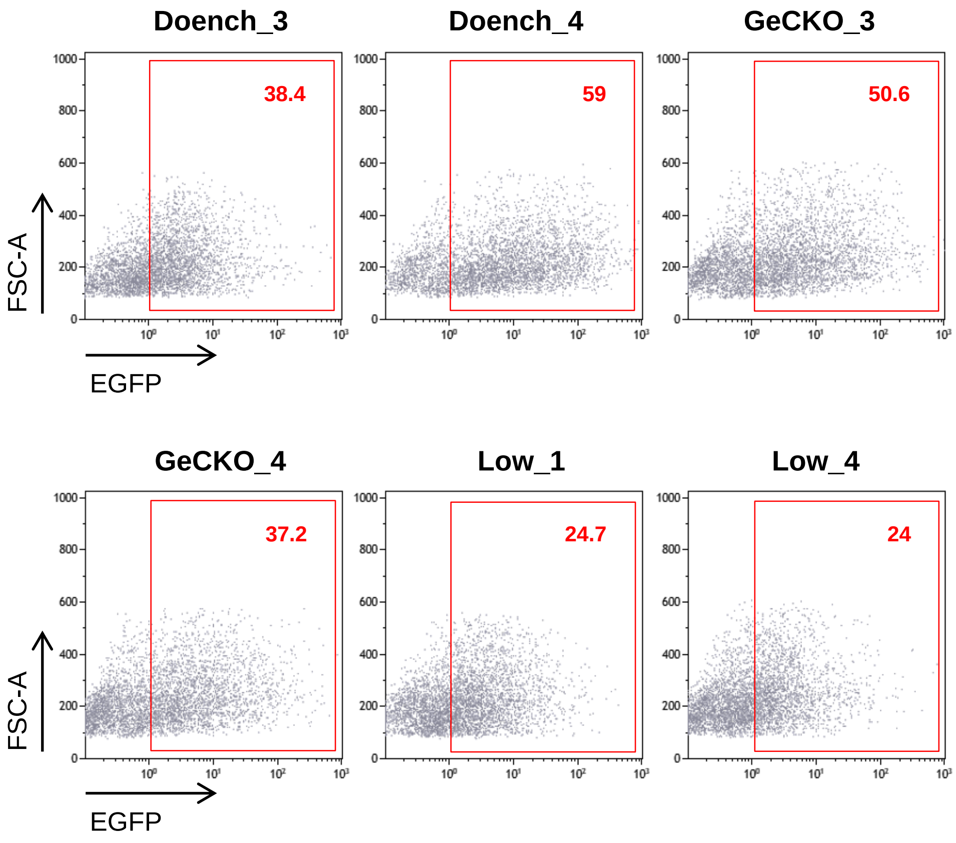

Supplemental Figure 7

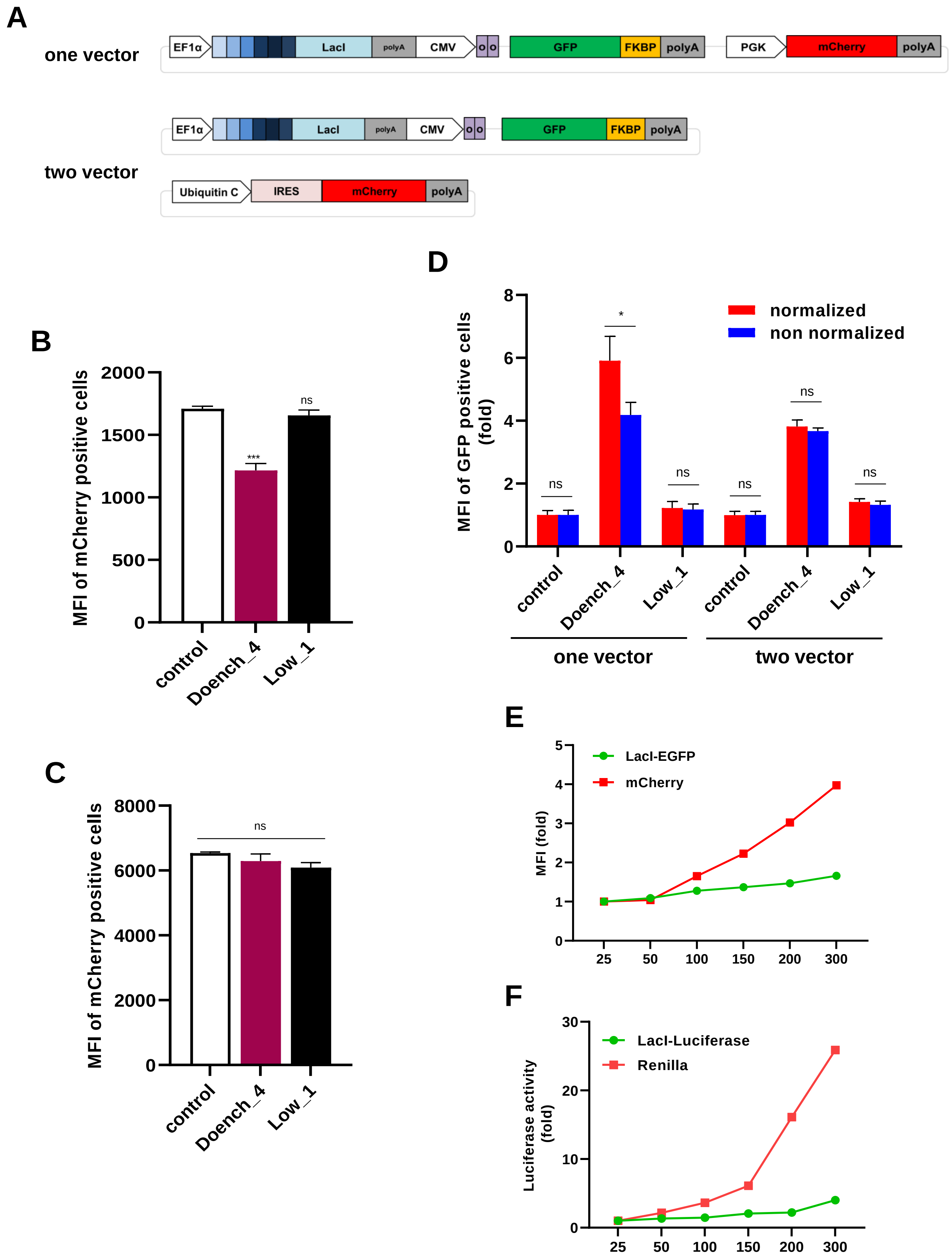

Supplement: gkab467_Supplemental_Files [file gkab467_supplemental_files.zip › Jung_et_al_Supplemental Figures.pdf]
